# Tai Chi versus conventional exercise for improving cognitive function in older adults: a pilot randomized controlled trial

**DOI:** 10.1038/s41598-022-12526-5

**Published:** 2022-05-25

**Authors:** Angus P. Yu, Edwin C. Chin, Danny J. Yu, Daniel Y. Fong, Calvin P. Cheng, Xiaoqing Hu, Gao X. Wei, Parco M. Siu

**Affiliations:** 1 Division of Kinesiology, School of Public Health, Li Ka Shing Faculty of Medicine, The University of Hong Kong, Pokfulam, Hong Kong, China; 2 School of Nursing, Li Ka Shing Faculty of Medicine, The University of Hong Kong, Pokfulam, Hong Kong, China; 3 Department of Psychiatry, Li Ka Shing Faculty of Medicine, The University of Hong Kong, Pokfulam, Hong Kong, China; 4 Department of Psychology, Faculty of Social Sciences, The University of Hong Kong, Pokfulam, Hong Kong, China; 5 Institute of Psychology, Chinese Academy of Sciences (CAS) Key Laboratory of Mental Health, Beijing, China

**Keywords:** Translational research, Neurodegenerative diseases

## Abstract

Studies have shown that Tai Chi and conventional exercise can modify the brain through distinct mechanisms, resulting in different brain adaptations. Therefore, it is conceivable to speculate that these two exercise modalities may have different effects on improving cognitive function. This study was a parallel group, assessor-blinded, pilot randomized controlled trial comparing the effects of Tai Chi and conventional exercise on improving cognitive function in older persons with mild cognitive impairment (MCI). A total of 34 adults aged ≥ 50 years with MCI were randomized (1:1:1) to the Tai Chi group (TC, *n* = 10, 3 sessions of 60-min Yang-style Tai Chi training per week for 24 weeks), conventional exercise group (EX: *n* = 12, 3 sessions of 60-min fitness training per week for 24 weeks), or control group (CON: *n* = 12, no intervention). Global cognitive function assessed by the Hong Kong version of the Montreal Cognitive Assessment (MoCA-HK) and performance in various cognitive domains were examined at baseline, and 12 and 24 weeks of the intervention. Both exercise groups showed improved global cognitive function as measured by MoCA-HK compared with the control group after 12 and 24 weeks of the intervention, (all *P* < 0.001). Only TC achieved clinically relevant improvement on global cognitive function at week 12. Both exercise groups achieved clinically relevant improvements at the end of the interventions at week 24. Compared with EX, TC exhibited greater improvements on global cognitive function indicated by MoCA-HK after 12 weeks of the intervention (*P* < 0.001) and cognitive flexibility indicated by part B/A ratio score of the Trail Making Test throughout the study (all *P* < 0.05). Both interventions were equally effective in improving the other examined cognitive domains. Further studies are needed to substantiate the superior long-term benefits of Tai Chi on global cognitive function compared with conventional exercise, and dissect the underlying mechanisms of the two exercises on improving cognitive domains and the corresponding brain adaptations.

* Trial registration*: This study was registered at clinicaltrials.gov (Trial registration number: NCT04248400; first registration date: 30/01/2020).

## Introduction

Dementia is characterized by a substantial decline in cognitive functions that adversely affects daily functioning^1^. Dementia most often affects older adults, with the risk of developing dementia increasing with age. The prevalence of dementia is expected to rise with an aging global population^2^, leading to increasing burdens on healthcare systems and on society. As there are currently no effective treatments for dementia, developing preventive strategies may be the only way to tackle this burgeoning disease^3^. Mild cognitive impairment (MCI) is a state that the cognitive function of an individual declines faster than normal aging but have not yet affected daily living. As an intermediate state between normal cognitive function and dementia, presence of MCI increases the chance of developing dementia, with approximately two-thirds of Alzheimer’s disease patients having a previous diagnosis of MCI^4^. Moreover, the reported 2-year cumulative dementia incidence in individuals > 65 years with MCI was alarmingly high at 14.9%^5^. Previous studies demonstrated that prompt lifestyle interventions in MCI patients can delay cognitive declines and maintain normal cognitive performance^6,7^. It is suggested that timely intervention within the period of MCI is critical to slow down or reverse the decline in cognitive function and ultimately delay or prevent the onset of dementia^4^.

Accumulating evidence has shown that exercise, including aerobic exercise^8^ and resistance exercise^9–11^, has beneficial effects on cognitive function. Indeed, both the World Health Organization and the American Association of Neurology suggest that physical exercise can reduce the risk of cognitive decline and the risk of developing dementia^5,12^. The practical guidelines of the American Association of Neurology suggest physical exercise twice a week for 6 months can improve cognitive function in individuals presenting with MCI, although there is no recommendation on the exercise modality^5^. An exercise modality that is low in cost, relatively safe with no side effects, and widely acceptable to the older population will be crucial to sustain regular exercise for preventing cognitive decline. Tai Chi is a popular mind–body exercise that incorporates meditation and aerobic exercise, which is in contrast to conventional exercises such as running, swimming, and resistance training that do not involve a meditation element. Moreover, Tai Chi is widely accepted to be a suitable exercise for older adults^13^. Recent meta-analyses have suggested that Tai Chi can delay cognitive decline in older adults with MCI^14^ and improve cognitive function in individuals with early stage dementia^15^. Collectively, Tai Chi may present a promising intervention to prevent cognitive decline in older adults with MCI.

It is conceivable that improvement in cognitive function is achieved by structural and functional changes in brain. Although ample evidence suggests both conventional exercise and Tai Chi have beneficial effects on cognitive function, these two exercise modalities are likely to improve cognitive function through distinct brain mechanisms^16^. Conventional exercise is known to improve cognitive function by increasing cardiovascular fitness, altering cerebral blood flow^17,18^ and enhancing neuroplasticity in a global manner. In contrast, Tai Chi induces improvements in neuroplasticity through its motor complexity and multiple components combined with meditation training, relaxation practice, and aerobic exercise^16^. Their distinct mechanisms of action result in different brain adaptions. Tai Chi has recently been shown to induce brain plasticity in a more robust manner than aerobic exercise^19^. More importantly, these exercise modalities are found to provoke different structural and functional changes in brain regions associated with executive function, memory, and visuospatial processing in healthy adults^19,20^. Common brain adaptation patterns are also found between individuals well-trained in Tai Chi and their counterparts practicing meditation or conventional exercise alone^21^. Meditation alone is beneficial to memory and cognitive flexibility, which is a subdomain of executive function^22^, hence it is conceivable that the meditation element of Tai Chi can augment the effects of the physical exercise element on various cognitive domains or subdomains. Revealed by a cross-sectional study, individuals with long-term practice of Tai Chi outperform their counterparts with regular conventional exercise in the neurocognitive tests associated with memory and executive function^23^. Taken together, the two exercise modalities are likely to confer different effects on improving global cognitive function and cognitive domains in older adults with cognitive decline. However, published intervention studies that compare directly between the effects of Tai Chi and conventional exercise on global cognition and specific cognitive domains are scarce. To the best of our knowledge, the present work is the first to reveal how cognitive function is respectively modulated by these exercise modalities in older adults with MCI. We hypothesize that Tai Chi and conventional exercise induce differential improvements on global cognitive function and on specific cognitive domains including memory and executive function, especially in the cognitive flexibility subdomain. As poor sleep quality and mood are risk factors of cognitive decline^24^ and impaired cognitive function negatively impacts quality of life^25^, we also monitored sleep quality, mood, and quality of life of participants during the study period. Besides neurocognitive outcomes, physical performance was also measured to assess the exercise training effects.

## Methods

### Study design and participants

This single-center, assessor-blinded, three-arm, parallel group, pilot randomized controlled trial was conducted at a single research site in Hong Kong. Participants were recruited through promotions in community centers, elderly day-care centers, housing estates, and local universities. Chinese adults ≥ 50 years of age with MCI defined by a score equal or below the 7th percentile of the normative data using the age- and education-corrected Hong Kong version of the Montreal Cognitive Assessment (MoCA-HK)^26,27^ and normal daily functioning score ≥ 2 marks (on a 4-point scale) in each item of the Chinese Lawton Instrumental Activities of Daily Living Scale^28^ were recruited from the Hong Kong community. Written informed consent was obtained before the start of the study. Individuals were excluded from the study if they were incapable of performing exercise; practiced regular mind–body exercise (> 3 times 60-min sessions per week) or physical exercise (> 150 min of moderate intensity physical activity or > 75 min of vigorous intensity physical activity weekly) over the past 6 months; or had a history of major diseases such as cancer, stroke, cardio-/cerebrovascular, neurodegenerative, and renal disease.

### Ethical approval

This study followed the principles of the Declaration of Helsinki. Ethical approval for the experimental procedures were obtained from the Institutional Review Board of the regional healthcare system in Hong Kong (IRB reference number: UW 18–454). This study was performed in accordance with the relevant guidelines and regulations of the Institutional Review Board.

### Sample size estimation and randomization

Based on a medium effect size of interaction Cohen’s d = 0.6, 10 participants per group were required to achieve an 80% statistical power (α = 0.05) for changes between intervention groups and the control group. Eligible participants were randomly allocated to the control (CON), conventional exercise (EX) and Tai Chi (TC) groups in a 1:1:1 ratio with a block size of 12. The computer-generated randomization sequence was prepared by an independent researcher. The randomization sequence was concealed from the researcher responsible for participant recruitment. The outcome assessors were blinded to the group allocation.

### Intervention

Participants allocated to the EX group received 24 weeks of conventional exercise training comprising three supervised 1-h group training sessions weekly. Each 1-h training session consisted of 10 min of warm-up static stretching exercises targeting the major muscle groups (i.e., neck lateral flexion, anterior cross-arm stretch, behind the neck triceps stretch, standing quadricep stretch with chair assist, seated toe touch stretch, and wall calf stretch), 20 min of muscle-strengthening exercises (standing lateral raise, standing arm curl, squat with chair assist, standing leg curl with chair assist, and calf raise with chair assist), 20 min of aerobic exercises (stepping with air shoulder press, stepping with arm swing, stepping with punching, stepping with arm abduction, stepping with arm curl, and stepping with shoulder rotation), followed by 10 min of cool-down stretching exercises (similar to the warm-up exercises). In the first 12 weeks, participants in the EX group performed muscle-strengthening and aerobic exercises without a load (i.e., unloaded bodyweight), whereas in the latter 12 weeks, exercises were performed with light loading using elastic bands and a 0.5 kg dumbbell. The conventional exercise training sessions were conducted by a certified fitness instructor. Details of the training protocol of the conventional exercise intervention is provided in Supplementary Table 1. Participants allocated to the TC group received a 24-week Tai Chi intervention incorporating the 24-form Yang-style Tai Chi and comprising three supervised 1-h group training sessions weekly. Each 1-h training session began with 10 min of standing pose meditation (i.e., Zhan Zhuang) and Tai Chi relaxation exercises (i.e., stretching with a meditation element), followed by 40 min of the Yang-style Tai Chi program, and concluding with 10 min of standing pose meditation and Tai Chi relaxation exercises. The Tai Chi training sessions were delivered by a certified Tai Chi instructor. Details of the training protocol of the Tai Chi intervention is provided in Supplementary Table 2. Both interventions were administered indoors in a room at the research site. Research personnel occasionally visited the training sessions to ensure the interventions were delivered according to the protocol. The exercise intensity of both interventions was comparable and considered to be at a moderate level according to the WHO’s recommendation for moderate-intensity physical activity (i.e., 3.0–5.9 metabolic equivalents [METs]; 1 MET refers to the resting metabolic rate during quiet sitting). The exercise intensity of the conventional exercise group was in the range of 4.3–5.5 METs according to the Compendium of Physical Activities (PA code 02035: conditioning exercise with moderate effort, 4.3 METs; PA code 03015: aerobic stepping, 5.5 METs)^29^. A previous study has used a direct measurement of metabolic expenditure to show that the exercise intensity of Yang-style Tai Chi was 3.24 METs^30^. Although training intensity was not objectively measured, instructors of both intervention groups continuously assessed the perceived intensity during the training session using the Rated Perceived Exertion Scale^31^. The training intensity in each intervention was regulated by the respective instructor by adjusting the pace, body movement, and body position of participants, such that the perceived exertion was maintained at approximately 13 on the Rate of Perceived Exertion scale from 6 to 20^31^. Participants allocated to the CON group received no intervention and were instructed to maintain their usual daily activities during the study period.

#### Outcomes and monitoring parameters

Outcome assessments were performed at baseline, after 12 weeks of the intervention (mid-assessment), and upon completion of the 24-week intervention (post-assessment). All assessments were conducted the same day in a quiet room. The assessors were blinded for group allocation. They had no roles in participant enrollment and randomization. Assessments for cognitive function were performed in the following order: (1) MoCA-HK (Verbal Fluency test from MoCA-HK), (2) 30-min Delay Recall Test, (3) Digit-Span Forward Test, (4) Digit-Span Backward Test, (5) Trail Making Test Part A, (6) Trail Making Test Part B, (7) Victoria Stroop Test (Dot condition), (8) Victoria Stroop Test (Word condition), (9) Victoria Stroop Test (Interference condition), and (10) N-back Task. Assessments for sleep, quality of life, and physical performance were performed after completion of the neurocognitive assessments in the following order: (1) Pittsburgh Sleep Quality Index, (2) 12-item Short Form Survey, (3) Five Times Chair Stand Test, and 4) Single Leg Stand Test. The whole assessment session lasted approximately 1 h and 15 min.

Considering that mild cognitive impairment and dementia are usually diagnosed by global cognitive performance, we selected MoCA-HK to assess global cognitive function as the primary outcome due to its high clinical relevance. A higher MoCA-HK score indicates better global cognitive function. Participants were considered to have reached a clinically relevant improvement in their global cognitive function if they showed an increase in MoCA-HK that exceeds the minimal clinically important difference which is defined as the minimal change that significantly attenuates a given disease condition^32^ (i.e., at least 4 marks compared with baseline)^33–35^. The secondary outcomes were changes in cognitive domains including executive function, memory, attention, and language. Long-term memory was assessed by the 30-min Delay Recall Test using the 10-word list from the Delay Recall Test section of the Chinese abbreviated mild cognitive impairment test^36^. The 30-min Delay Recall Test started with three learning trials. In each trial, the assessor would read out a 10-word list and participants were immediately asked to recall the 10 words. After completion of the learning trials, participants were asked to recall the 10 words after 30 min. The number of words successfully recalled by participants was recorded. Short-term memory and working memory were assessed using the forward and backward subsets of the Digit Span Test, respectively^37^. The backward span test was performed followed by the completion of the forward span test. In each test, the assessor would read out a sequence of numbers at a speed of ~ 1 digit per second. Participants were asked to repeat the sequence in the forward span in the same order and repeat the backward span in the reverse order. There were two trials at each level and the difficulty was increased by adding an extra digit at each level. Participants were required to complete both trials even if they did not successfully recall the sequence in the first trial. The forward span began with a 3-digit sequence whereas the backward span started with a 2-digit sequence. The test was terminated when participants failed both trials at the same difficulty level. The maximum number of digits in the sequence that participants successfully recalled and the total number of successful trials were recorded as the length and span scores. While the N-back task usually considered as an assessment of working memory, a recent study demonstrated that the performance of the N-Back task in older population was associated with attention, verbal memory, updating, and executive processes^38^. Therefore, the N-back task was used to assess attention, executive function in addition to a unitary working memory function. The N-back Task was administered by computer using the Psychology Experiment Building Language (PEBL) test battery^39^. Participants were presented with a series of visual stimuli and asked to response whether a stimulus matched a stimulus 1 trial before in the 1-back condition and 2 trials before in the 2-back condition. The correctness of each response and the reaction time in each trial were recorded.

Executive function was assessed by the Trail Making Test (TMT) using the paper version of a modified protocol^40,41^. The modified TMT consisted of two parts: Part A reflects visual perception performance^42^ and included nine circled Arabic numbers randomly arranged on the test sheet, whereas Part B reflects working memory and task-switching performance^42^ and included nine circled Arabic numbers and nine Chinese numbers randomly arranged on the test sheet. Participants were required to connect all the numbers in ascending order in Part A (i.e., 1 → 2 → 3 → 4 → 5…) and alternate Arabic and Chinese numbers in ascending order in Part B (i.e.. 1 → 一 → 2 → 二 → 3 → 三 → 4 → 四 → 5 → 五…) as quickly as possible without lifting the pen from the paper. The time required for participants to finish connecting all dots in each part was recorded. Participants were given a trial run to familiarize them with each part of the test before actual testing. The Part B-A difference score is indicative of executive control, which is the ability to execute goal-directed behaviors by orchestrating complex mental processes and cognitive abilities^43^. The Part B-A difference score was calculated by subtracting the time required to complete Part A from that of Part B^42^. To calculate the Part B/A ratio score, a surrogate of cognitive flexibility, which is the ability to selectively switch between mental processes to generate appropriate behavioral responses and achieve efficient adaptation in task-changing challenges^44,45^, the time to complete Part B was divided by that of Part A^46,47^. Executive function consists of executive control and cognitive flexibility but regulation of these subdomains do not necessarily share the same neural patterns or inter-regions connectivity^45^. it has been proposed that the specific computations (i.e., activity and connectivity) among brain regions determine which kind of executive function (i.e., executive control and cognitive flexibility) is behaviorally observed.

Attention and language ability were assessed using the Chinese version of the Victoria Stroop Test^48^ and Verbal Fluency Test, respectively. The Victoria Stroop Test consists of three subtasks with different conditions: the dot condition, the word condition, and the interference condition. The stimuli in each condition were printed on A4 paper in blue, green, red, or yellow. The stimuli of the dot condition consisted of dots, the stimuli of the word condition consisted of common words unrelated to the concept of colors, and the stimuli of the interference condition consisted of the actual names of the colors (i.e., blue, green, red, and yellow). Each card contained six rows of four items in the four colors arranged in a pseudo-random order within the array. Each of the four colors was used six times, with each color appearing once per row. Participants were asked to name the color of the ink that the stimulus was printed in, while also disregarding the word content. The completion time and number of errors in each condition were recorded. In the Verbal Fluency Test, participants were asked to name as many animals as possible in 1 min. The number of non-repeated animals was recorded.

Muscle strength, balance, health-related quality of life, and sleep quality were monitored during the study period. Lower limb muscle strength was examined by the Five Times Chair Stand Test^49^. Balance was assessed by the Single Leg Stand Test with the eyes open^50^. The health-related quality of life, mood, and subjective sleep quality were assessed by the 12-item Short Form Survey (SF-12)^51^, Hospital Anxiety and Depression Scale (HADS)^52^, and Pittsburgh Sleep Quality Index (PSQI)^53^, respectively.

### Adverse events

At the end of each training session, instructors asked the participants if there were any adverse events. The research personnel responsible for making the assessment appointments also asked the participants if there were any adverse events during the phone call for making assessment appointment and after each assessment session.

### Statistical analysis

Data were expressed as mean (SD) and analyzed using generalized estimating equations with baseline, group, time, and the group-by-time interaction as covariates using the R package ”geeM”. A significant group-by-time interaction indicated a difference in the intervention-mediated changes among interventions by time. Pairwise comparisons with baseline adjustment were then performed with R package “multcomp” using a closed test procedure with Bonferroni-Holm correction^54,55^. Statistical significance was considered at *P* < 0.05. All statistical analyses were performed using R (Version 4.0.1).

The effect size is expressed as Cohen’s d. The between group effect size, refers to the difference in within group effect size between the two groups at a given time point, are presented. To calculate the within group effect size of a group, the mean at baseline was subtracted from its counterpart at a time point of interest, and then divided by the pooled standard deviation of the two time points. The formula for calculating Cohen’s d and pooling two standard deviation are presented below:

$${\text{Cohen}}^{\prime } {\text{s d}} = \frac{{Mean_{{\left( {assessment{\kern 1pt} time{\kern 1pt} point} \right)}} - Mean_{{\left( {baseline} \right)}} }}{{SD_{{pooled}} }}$$

Formula for pooling two standard deviations: $SD_{pooled} = \sqrt {\frac{{(SD_{1}^{2} + { }SD_{2}^{2} ) }}{2}}$.

## Results

**Figure 1:**
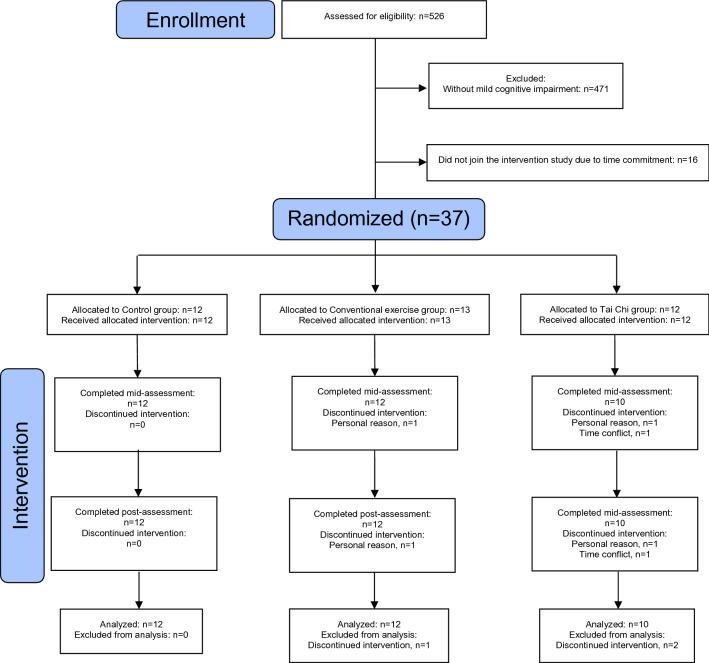
Schematic Presentation of Participant Screening, Randomization, and Interventions.

Participant recruitment was conducted from October 2018 to August 2019. The baseline characteristics of participants are summarized in Table 1. After screening 526 recruits, 37 out of 53 eligible participants agreed to participate in our study (Fig. 1). They were randomly assigned to CON (*n* = 12), EX (*n* = 13), and TC (*n* = 12) groups. The interventions were started within 1 month after the baseline assessment. All participants in the CON group also participated in the mid- and post-assessments. One participant in the EX group dropped out due to personal reasons and did not participate in any further assessments. Two participants in the TC group did not attend the Tai Chi training or subsequent assessments because of personal reasons or time conflicts. Data collection was completed by February 2020 and no adverse events were recorded in the study. The baseline characteristics of the three groups are similar. Given that clinical relevant difference, by definition, requires a minimum difference of 4 points in MoCA-HK^33–35^, there were no clinically relevant difference observed in the baseline global cognitive function among the studied groups. The attendance rate (i.e., percentage of training session participated) in the two intervention groups were similar (TC: 80.6% vs. EX: 77.5%).

**Table 1:** Baseline characteristics of participants.

	CON	EX	TC
Number of participants, n	12	12	10
Gender, F:M	10:2	8:4	7:3
Age, year	67.6 (8.1)	67.2 (6.8)	67.3 (4.2)
Height, cm	156.2 (6.0)	157.7 (9.0)	155.2 (9.0)
Weight, kg	59.9 (8.7)	55.9 (8.7)	58.1 (11.0)
Years of education, year	10.9 (3.7)	11.4 (3.8)	11.8 (2.4)
**International physical activity questionnaire**			
Activity, metabolic equivalent-min/week	773.4 (111.3)	749.8 (137.7)	806.3 (84.0)
Sitting, metabolic equivalent-min/week	2940.0 (877.4)	2660.0 (944.8)	2415.0 (544.5)
**Marriage status, *n***			
Never married	0	0	1
Married	8	11	6
Widowed	3	0	2
Divorced	1	1	1
Smoker, n	0	0	0
Family history of dementia, n	1	1	2

All values are expressed as mean (SD). *CON* Control group, *EX* Conventional exercise group, *TC* Tai Chi group. A clinically relevant difference in global cognitive function between groups was considered if the MoCA-HK score was differed by the minimal clinical important difference (i.e. the MoCA-HK score differs by at least 4 points^33–35^). The habitual physical activity of the participants was assessed by the International Physical Activity Questionnaire^70^. The baseline characteristics of the three groups were similar and there was no clinically relevant difference in global cognitive function at baseline.

### Primary outcome

Both TC and EX groups showed significant improvements in MoCA-HK scores compared with CON at both mid- and post-assessments (both *P* < 0.001). At mid-assessment, TC showed greater improvements in MoCA-HK than EX (*P* < 0.001). At post-assessment, TC tended to show greater improvements in MoCA-HK than EX, but this did not reach statistical significance (*P* = 0.061) (Table 2).

**Table 2:** Summary of neurocognitive assessments.

	Baseline (0-week)	Mid (12-week)	Post (24-week)	Group-by-time Interaction effect	Group effect	Time effect		Mid			Post		
								ΔMean_adj_ [95%CI]	P-value	Cohen’s d	ΔMean_adj_ [95%CI]	P-value	Cohen’s d
**Primary outcome**													
**Hong Kong version of montreal cognitive assessment**													
CON	18.2 (3.8)	18.5 (4.7)	18.9 (5.2)	< 0.001	< 0.001	0.029	EX vs. CON:	2.2 [1.0 to 3.4]	< 0.001	1.23	4.7 [3.5 to 5.9]	< 0.001	2.36
EX	19.3 (2.0)	22.1 (2.3)	25.0 (2.5)				TC vs. CON:	4.4 [3.2 to 5.7]	< 0.001	2.61	6.0 [4.8 to 7.3]	< 0.001	3.88
TC	19.7 (1.5)	24.6 (2.1)	26.6 (1.9)				TC vs. EX:	2.2 [1.0 to 3.5]	< 0.001	1.39	1.3 [0.04 to 2.5]	0.061	1.51
**Secondary outcomes**													
**Trail making test**													
*Part A, s*													
CON	18.4 (9.5)	16.7 (10.3)	16.0 (9.8)	0.724	0.164	0.035	EX vs. CON:	− 3.8 [ − 7.5 to − 0.1]	0.214	− 0.37	− 3.6 [ − 7.3 to 0.1]	0.214	− 0.38
EX	15.9 (11.1)	11.2 (5.4)	10.7 (3.8)				TC vs. CON:	− 1.3 [ − 5.2 to 2.6]	0.701	− 0.09	− 0.7 [ − 4.6 to 3.2]	0.800	− 0.04
TC	13.4 (6.4)	12.0 (4.0)	11.9 (3.4)				TC vs. EX:	2.5 [ − 1.4 to 6.3]	0.475	0.28	2.9 [ − 1.0 to 6.8]	0.366	0.33
*Part B, s*													
CON	64.8 (34.4)	63.1 (31.1)	73.3 (47.6)	0.011	0.101	0.174	EX vs. CON:	− 12.7 [ − 29.7 to 4.3]	0.289	− 0.33	− 31.5 [ − 48.5 to − 14.5]	0.005	− 0.91
EX	57.1 (38.3)	45.9 (17.0)	37.2 (11.0)				TC vs. CON:	− 17.5 [ − 35.3 to 0.3]	0.139	− 0.67	− 33.8 [ − 51.6 to − 16.0]	0.005	− 1.22
TC	60.0 (30.1)	42.8 (15.1)	36.7 (12.5)				TC vs. EX:	− 4.8 [ − 22.7 to 13.0]	0.764	− 0.34	− 2.3 [ − 20.1 to 15.5]	0.919	− 0.30
*Part B-A difference score*													
CON	46.4 (30.7)	46.4 (22.5)	57.3 (38.0)	0.005	0.058	0.094	EX vs. CON:	− 8.9 [ − 23.2 to 5.5]	0.354	− 0.29	− 27.9 [ − 42.2 to − 13.5]	0.003	− 1.02
EX	41.2 (28.0)	34.6 (16.2)	26.5 (9.1)				TC vs. CON:	− 15.8 [ − 30.8 to − 0.7]	0.111	− 0.78	− 32.6 [ − 47.7 to − 17.6]	0.001	− 1.43
TC	46.7 (25.5)	30.8 (13.2)	24.8 (10.9)				TC vs. EX:	− 6.9 [ − 22.0 to 8.2]	0.493	− 0.49	− 4.8 [ − 19.8 to 10.3]	0.663	− 0.41
*Part B/A ratio score*													
CON	3.8 (1.7)	4.0 (1.0)	4.5 (0.6)	< 0.001	0.007	0.003	EX vs. CON:	0.4 [ − 0.2 to 1.1]	0.223	0.45	− 0.8 [ − 1.4 to − 0.2]	0.030	− 0.66
EX	3.7 (0.9)	4.4 (1.4)	3.6 (0.9)				TC vs. CON:	− 0.8 [ − 1.5 to − 0.2]	0.032	− 0.84	− 1.9 [ − 2.5 to − 1.2]	< 0.001	− 1.62
TC	4.7 (1.7)	3.7 (1.1)	3.2 (1.0)				TC vs. EX:	− 1.3 [ − 1.9 to − 0.6]	< 0.001	− 1.29	− 1.1 [ − 1.7 to − 0.4]	0.005	− 0.96
**30-min Delay Recall Test, number of words recalled**													
CON	4.2 (2.7)	5.0 (3.1)	4.6 (3.3)	< 0.001	0.060	0.594	EX vs. CON:	1.4 [0.2 to 2.5]	0.036	0.43	2.9 [1.7 to 4.0]	< 0.001	1.10
EX	4.8 (3.0)	6.8 (2.7)	7.9 (1.9)				TC vs. CON:	1.8 [0.6 to 3.0]	0.008	0.79	2.8 [1.6 to 4.0]	< 0.001	1.46
TC	5.2 (2.2)	7.6 (2.3)	8.2 (1.5)				TC vs. EX:	0.4 [ − 0.8 to 1.6]	0.618	0.37	− 0.1 [ − 1.3 to 1.1]	0.895	0.36
**Digit span forward**													
*Length, number of digits*													
CON	7.3 (0.9)	7.4 (1.0)	6.6 (1.0)	0.003	0.025	0.006	EX vs. CON:	0.6 [0.02 to 1.1]	0.102	0.59	1.5 [0.9 to 2.0]	< 0.001	1.55
EX	7.5 (1.0)	8.1 (0.7)	8.2 (0.7)				TC vs. CON:	0.4 [ − 0.1 to 1.0]	0.254	0.36	1.2 [0.6 to 1.7]	< 0.001	1.06
TC	7.7 (0.7)	8.1 (1.0)	8.0 (1.1)				TC vs. EX:	− 0.1 [ − 0.7 to 0.4]	0.854	− 0.23	− 0.3 [ − 0.9 to 0.3]	0.425	− 0.49
*Mark, number of correct trials*													
CON	9.7 (1.4)	9.4 (1.6)	8.4 (1.4)	0.001	0.047	0.012	EX vs. CON:	1.6 [0.7 to 2.5]	0.002	1.06	3.0 [2.1 to 3.9]	< 0.001	1.95
EX	9.8 (1.9)	11.1 (1.0)	11.5 (1.4)				TC vs. CON:	1.3 [0.4 to 2.3]	0.015	0.62	2.3 [1.4 to 3.3]	< 0.001	1.34
TC	10.6 (1.8)	11.4 (2.0)	11.4 (2.1)				TC vs. EX:	− 0.3 [ − 1.2 to 0.7]	0.627	− 0.44	− 0.7 [ − 1.6 to 0.3]	0.218	− 0.61
**Digit span backward**													
*Length, number of digits*													
CON	4.0 (1.1)	3.9 (1.7)	4.3 (1.9)	0.216	0.389	0.875	EX vs. CON:	0.7 [ − 0.1 to 1.5]	0.308	0.67	0.9 [0.1 to 1.7]	0.112	0.95
EX	4.0 (1.0)	4.6 (1.0)	5.2 (1.1)				TC vs. CON:	0.3 [ − 0.5 to 1.2]	0.718	0.30	0.7 [ − 0.1 to 1.6]	0.308	0.72
TC	4.3 (0.7)	4.5 (1.0)	5.2 (1.2)				TC vs. EX:	− 0.3 [ − 1.2 to 0.5]	0.749	− 0.37	− 0.2 [ − 1.0 to 0.6]	0.826	− 0.23
*Mark, number of correct trials*													
CON	4.8 (2.1)	5.3 (3.1)	5.7 (3.2)	0.095	0.130	0.556	EX vs. CON:	0.4 [ − 0.8 to 1.6]	0.637	0.24	1.0 [ − 0.2 to 2.2]	0.269	0.59
EX	5.5 (1.8)	6.3 (1.9)	7.3 (2.1)				TC vs. CON:	0.5 [ − 0.7 to 1.8]	0.581	0.63	1.1 [ − 0.1 to 2.4]	0.269	1.24
TC	5.2 (1.0)	6.2 (1.4)	7.2 (1.5)				TC vs. EX:	0.1 [ − 1.1 to 1.4]	0.953	0.39	0.1 [ − 1.1 to 1.4]	0.953	0.65
**N-back tasks**													
*1-back correction rate, %*													
CON	73.5 (8.2)	72.7 (10.3)	70.5 (9.6)	0.003	0.001	0.008	EX vs. CON:	2.4 [ − 3.8 to 8.5]	0.748	0.55	6.1 [ − 0.03 to 12.3]	0.332	1.05
EX	70.5 (5.7)	73.5 (7.2)	75.0 (6.9)				TC vs. CON:	− 2.2 [ − 8.8 to 4.4]	0.748	0.09	8.3 [1.7 to 14.8]	0.206	1.18
TC	67.3 (8.8)	67.3 (9.8)	75.5 (10.5)				TC vs. EX:	− 4.5 [ − 11.0 to 1.9]	0.469	− 0.46	2.1 [ − 4.3 to 8.6]	0.748	0.14
*1-back reaction time, ms*													
CON	755.4 (255.7)	712.6 (172.0)	702.9 (245.6)	0.771	0.985	0.542	EX vs. CON:	32.4 [ − 142.0 to 206.8]	0.969	0.06	46.3 [ − 131.9 to 224.5]	0.969	0.08
EX	822.3 (410.4)	776.1 (247.8)	774.7 (326.9)				TC vs. CON:	18.3 [ − 164.4 to 201.0]	0.969	− 0.08	60.7 [ − 125.7 to 247.1]	0.969	0.10
TC	803.6 (221.9)	753.4 (136.1)	780.4 (183.2)				TC vs. EX:	− 14.1 [ − 196.7 to 168.5]	0.969	− 0.14	14.4 [ − 168.2 to 197.0]	0.969	0.01
*2-back correction rate, %*													
CON	68.1 (7.0)	67.4 (9.7)	64.6 (6.3)	0.014	0.035	0.086	EX vs. CON:	0.3 [ − 4.9 to 5.5]	0.942	0.08	8.5 [3.3 to 13.7]	0.053	1.19
EX	67.4 (7.5)	67.4 (7.5)	72.8 (8.6)				TC vs. CON:	− 1.5 [ − 7.0 to 4.0]	0.885	− 0.08	5.5 [ − 0.01 to 11.0]	0.260	1.18
TC	65.8 (6.1)	65.0 (3.5)	69.2 (4.0)				TC vs. EX:	− 1.7 [ − 7.2 to 3.7]	0.836	− 0.16	− 3.0 [ − 8.5 to 2.5]	0.714	− 0.01
*2-back reaction time, ms*													
CON	934.8 (189.3)	1012.3 (410.0)	851.4 (214.3)	0.493	0.423	0.427	EX vs. CON:	− 93.9 [ − 338.6 to 150.9]	0.961	− 0.12	52.4 [ − 186.0 to 290.9]	0.961	0.49
EX	843.8 (368.1)	882.2 (251.6)	877.3 (467.7)				TC vs. CON:	− 166.0 [ − 428.6 to 96.6]	0.961	− 0.81	147.9 [ − 98.1 to 394.0]	0.961	0.52
TC	1035.5 (300.6)	882.8 (229.4)	1077.9 (467.5)				TC vs. EX:	− 72.2 [ − 325.2 to 180.9]	0.961	− 0.69	95.5 [ − 156.7 to 347.7]	0.961	0.03
**Victoria stroop test**													
*Dot condition-completion time, s*													
CON	22.9 (10.3)	23.8 (12.2)	23.4 (17.4)	0.443	0.850	0.907	EX vs. CON:	− 5.5 [ − 10.8 to − 0.2]	0.242	− 0.59	− 8.3 [ − 13.6 to − 3.0]	0.029	− 0.89
EX	23.9 (12.3)	19.0 (5.8)	15.8 (5.3)				TC vs. CON:	− 3.5 [ − 9.1 to 2.1]	0.491	− 0.24	− 4.1 [ − 9.7 to 1.5]	0.423	− 0.35
TC	17.0 (8.0)	16.0 (3.6)	15.0 (4.3)				TC vs. EX:	2.0 [ − 3.6 to 7.6]	0.693	0.35	4.2 [ − 1.4 to 9.8]	0.417	0.54
*Dot condition-number of errors*													
CON	0.8 (1.2)	0.8 (1.1)	0.9 (1.8)	0.442	0.862	0.965	EX vs. CON:	− 0.4 [ − 1.0 to 0.2]	0.475	− 0.37	− 0.8 [ − 1.4 to − 0.2]	0.200	− 0.67
EX	0.9 (1.6)	0.4 (1.0)	0.2 (0.4)				TC vs. CON:	− 0.3 [ − 1.0 to 0.3]	0.609	− 0.71	− 0.5 [ − 1.1 to 0.1]	0.424	− 0.77
TC	0.2 (0.4)	0.0 (0.0)	0.0 (0.0)				TC vs. EX:	0.05 [ − 0.6 to 0.7]	0.930	− 0.33	0.3 [ − 0.3 to 0.9]	0.628	− 0.11
*Word condition-completion time, s*													
CON	31.3 (14.6)	30.0 (18.1)	31.5 (15.6)	0.298	0.851	0.947	EX vs. CON:	− 3.4 [ − 9.5 to 2.6]	0.481	− 0.21	− 8.8 [ − 14.8 to − 2.7]	0.070	− 0.59
EX	32.7 (17.7)	27.6 (17.9)	23.9 (12.6)				TC vs. CON:	− 3.9 [ − 10.3 to 2.4]	0.456	− 0.31	− 4.4 [ − 10.7 to 2.0]	0.456	− 0.24
TC	25.7 (14.9)	21.4 (5.0)	22.6 (12.0)				TC vs. EX:	− 0.5 [ − 6.9 to 5.9]	0.971	− 0.10	4.4 [ − 2.0 to 10.8]	0.456	0.34
*Word condition-number of errors*													
CON	1.3 (1.5)	0.8 (1.3)	0.9 (1.4)	0.375	0.125	0.126	EX vs. CON:	− 0.5 [ − 1.1 to 0.1]	0.311	− 0.19	− 0.3 [ − 0.8 to 0.3]	0.639	− 0.05
EX	1.0 (2.0)	0.2 (0.6)	0.5 (0.9)				TC vs. CON:	− 0.2 [ − 0.8 to 0.4]	0.665	− 0.06	0.1 [ − 0.5 to 0.7]	0.821	0.28
TC	0.6 (1.3)	0.2 (0.4)	0.6 (0.8)				TC vs. EX:	0.3 [ − 0.3 to 0.9]	0.637	0.13	0.4 [ − 0.2 to 1.0]	0.536	0.32
*Interference condition-completion time, s*													
CON	45.3 (19.4)	50.8 (25.0)	46.7 (23.3)	0.004	0.173	0.457	EX vs. CON:	− 15.5 [ − 25.5 to − 5.6]	0.015	− 0.67	− 16.6 [ − 26.6 to − 6.7]	0.012	− 0.63
EX	56.9 (35.3)	44.4 (22.9)	39.2 (27.1)				TC vs. CON:	− 7.6 [ − 18.0 to 2.7]	0.282	− 0.31	− 12.6 [ − 23.0 to − 2.3]	0.055	− 0.67
TC	42.3 (21.0)	40.8 (23.3)	31.7 (12.9)				TC vs. EX:	7.9 [ − 2.5 to 18.4]	0.274	0.35	4.0 [ − 6.4 to 14.4]	0.651	− 0.05
*Interference condition-number of errors*													
CON	2.3 (1.8)	2.8 (1.9)	2.8 (2.5)	0.021	0.142	0.180	EX vs. CON:	− 1.1 [ − 2.3 to 0.1]	0.300	− 0.54	− 1.5 [ − 2.7 to − 0.3]	0.170	− 0.68
EX	2.5 (2.6)	1.9 (1.7)	1.5 (1.7)				TC vs. CON:	− 1.3 [ − 2.6 to − 0.1]	0.249	− 0.76	− 1.5 [ − 2.8 to − 0.3]	0.170	− 0.87
TC	2.9 (2.3)	1.9 (1.7)	1.7 (1.3)				TC vs. EX:	− 0.3 [ − 1.5 to 1.0]	0.835	− 0.22	− 0.04 [ − 1.3 to 1.2]	0.983	− 0.19
**Verbal Fluency Test, number of animals**													
CON	14.6 (3.6)	15.1 (4.7)	15.1 (3.3)	0.164	0.750	0.739	EX vs. CON:	0.2 [ − 1.9 to 2.3]	0.991	− 0.04	0.8 [ − 1.4 to 2.9]	0.913	0.04
EX	15.9 (5.0)	16.3 (5.0)	16.8 (4.7)				TC vs. CON:	1.8 [ − 0.5 to 4.0]	0.410	0.55	2.2 [ − 0.1 to 4.4]	0.366	0.62
TC	16.4 (3.2)	18.2 (2.1)	18.6 (2.5)				TC vs. EX:	1.6 [ − 0.6 to 3.8]	0.472	0.59	1.4 [ − 0.8 to 3.6]	0.584	0.58

All values are expressed as mean (SD). Generalized estimating equations with baseline measurement as a covariate was used to analyze the data. Pairwise comparisons were performed using closed test procedure with Holm-Bonferroni correction. *CON* Control group, *EX* Conventional exercise group, *TC* Tai Chi group.

### Secondary outcomes

The cognitive performances are summarized in Table 2. Overall, there were no significant differences in TMT Part A scores. At mid-assessment, there were no significant differences in TMT Part B and Part B-A difference scores. At post-assessment, both interventions elicited robust improvements in TMT Part B scores (TC vs. CON: *P* = 0.005; EX vs. CON: *P* = 0.005) and Part B-A difference scores (TC vs. CON: *P* = 0.001; EX vs. CON: *P* = 0.003) compared with CON. However, there were no significant differences in the improvements in Part B and Part B-A difference scores between interventions groups. At mid-assessment, there was no significant difference in the Part B/A ratio score between EX and CON, whereas the decrease in the Part B/A ratio score in TC was more pronounced than in EX and CON (TC vs. EX: *P* < 0.001; TC vs. CON: *P* = 0.032). At post-assessment, the improvements in the Part B/A ratio scores in both intervention groups were more robust than in CON (TC vs. CON: *P* < 0.001; EX vs. CON: *P* = 0.030), with the improvements in TC more evident than in EX (*P* = 0.005).

Both interventions were associated with significant improvements in the 30-min Delay Recall Test compared with CON at mid-assessment (TC vs. CON: *P* = 0.008; EX vs. CON: *P* = 0.036) and at post-assessment (TC vs. CON: *P* < 0.001; EX vs. CON: *P* < 0.001), but there was no significant difference between intervention groups. There was no significant difference in the length of the Digit Span Forward at mid-assessment. However, both EX and TC demonstrated profound improvements in the length of the Digit Span Forward compared to CON at post-assessment (TC vs. CON: *P* < 0.001; EX vs. CON: *P* < 0.001). There were increases in the score of the Digit Span Forward in both EX and TC compared with CON at mid-assessment (TC vs. CON: *P* = 0.015; EX vs. CON: *P* = 0.002) and post-assessment (TC vs. CON: *P* < 0.001; EX vs. CON: *P* < 0.001), but there was no significant difference between intervention groups. There were also no significant differences in the length and score of the Digit Span Backward. There were no significant differences in the reaction time and correction rate of either the 1-back or 2-back test of the N-back Tasks.

There were no significant differences in the number of errors and the completion time of the Victoria Stroop Test under the dot and word conditions. There was also no significant difference in the number of errors in the Victoria Stroop Test under the interference condition. At mid-assessment, EX but not TC showed a significant improvement in the completion time of the Victoria Stroop Test under the interference condition compared with CON (*P* = 0.015), but this improvement did not reach statistical significance relative to TC. At post-assessment, EX exhibited a significant reduction in the completion time of the Victoria Stroop Test under the interference condition compared with CON (*P* = 0.012), with TC also showing a similar trend (*P* = 0.055), but there was no significant difference between intervention groups. Overall, there were no significant differences in the Verbal Fluency Test.

The results of the physical performance, mood, quality of life, and sleep assessments are summarized in Table 3. The improvements in the Five Times Chair Stand Test in EX and TC were more pronounced than in CON at both mid- and post-assessments (all *P* < 0.001), but there was no significant difference between the two intervention groups. There was no significant difference in left Single Leg Stand Test. At mid-assessment, there was a greater improvement in the right Single Leg Stand Test in TC but not in EX compared with CON (*P* = 0.034), although there was no statistical difference between the two intervention groups. At post-assessment, both TC and EX showed more improvements in the right Single Leg Stand Test compared with CON (TC vs. CON: *P* = 0.006; EX vs. CON: *P* = 0.019), but there was no significant difference between the two intervention groups. At mid-assessment, there was no significant difference in the anxiety score of HADS. At post-assessment, both intervention groups exhibited significant improvements compared with CON (TC vs. CON: *P* = 0.004; EX vs. CON: *P* = 0.010), but there was no significant difference between the intervention groups. Both interventions led to significant reductions in the depression score of HADS relative to CON at mid-assessment (TC vs. CON: *P* = 0.007; EX vs. CON: *P* = 0.030) and post-assessment (TC vs. CON: *P* = 0.006; EX vs. CON: *P* = 0.006), but there were no significant differences between the intervention groups over the course of study. At mid-assessment, the physical component score of SF-12 did not differ among all groups. At post-assessment, both intervention groups showed significant improvements in the physical component score of SF-12 compared with CON (TC vs. CON: *P* = 0.008; EX vs. CON: *P* = 0.049), but there was no significant difference between the two intervention groups. No significant differences in the mental component summary of the SF-12 and PSQI were observed.

**Table 3:** Summary of physical performance, mood, quality of life, and sleep assessments.

	Baseline (0-week)	Mid (12-week)	Post (24-week)	Group-by-time Interaction effect	Group effect	Time effect		Mid			Post		
								ΔMean_adj_ [95%CI]	*P*-value	Cohen’s d	ΔMean_adj_ [95%CI]	*P*-value	Cohen’s d
**Physical Performance**													
**Five Times Chair Stand Test, s**													
CON	10.4 (2.7)	11.5 (2.6)	11.3 (3.3)	< 0.001	0.125	0.067	EX vs. CON:	− 3.8 [− 5.2 to − 2.4]	< 0.001	− 1.39	− 4.5 [− 5.9 to − 3.1]	< 0.001	− 1.72
EX	10.5 (3.4)	7.7 (2.2)	6.9 (1.1)				TC vs. CON:	− 3.5 [− 5.0 to − 2.1]	< 0.001	− 1.97	− 3.8 [− 5.3 to − 2.4]	< 0.001	− 2.14
TC	9.7 (1.6)	7.5 (1.2)	7.1 (1.2)				TC vs. EX:	0.3 [− 1.2 to 1.7]	0.779	− 0.58	0.7 [− 0.8 to 2.1]	0.450	− 0.41
**Single Leg Stand Test**													
*Left leg, s*													
CON	38.5 (50.4)	35.1 (35.5)	37.0 (45.2)	0.102	0.074	0.838	EX vs. CON:	8.3 [− 12.7 to 29.3]	0.878	0.20	20.0 [− 1.0 to 41.0]	0.263	0.43
EX	61.2 (48.2)	67.0 (49.2)	80.6 (49.8)				TC vs. CON:	− 0.8 [− 22.6 to 21.0]	0.974	0.07	18.5 [− 3.3 to 40.3]	0.317	0.54
TC	46.6 (40.2)	46.2 (33.7)	67.4 (42.3)				TC vs. EX:	− 9.1 [− 30.9 to 12.7]	0.878	− 0.13	− 1.5 [− 23.3 to 20.3]	0.974	0.11
*Right leg, s*													
CON	44.3 (47.1)	44.0 (42.2)	40.6 (43.2)	< 0.001	0.005	0.052	EX vs. CON:	20.6 [0.2 to 40.9]	0.101	0.36	30.2 [9.9 to 50.6]	0.019	0.56
EX	56.2 (51.3)	74.8 (55.0)	81.0 (51.5)				TC vs. CON:	28.9 [7.6 to 50.2]	0.034	0.84	41.1 [19.8 to 62.4]	0.006	1.13
TC	45.8 (28.1)	74.2 (39.5)	83.0 (41.7)				TC vs. EX:	8.3 [− 13.0 to 29.6]	0.668	0.48	10.8 [− 10.5 to 32.2]	0.522	0.56
**Mood**													
**Hospital Anxiety and Depression Scale**													
*Anxiety score*													
CON	2.4 (2.5)	3.0 (3.6)	3.7 (3.4)	0.001	0.014	0.012	EX vs. CON:	− 0.9 [− 2.5 to 0.7]	0.444	− 0.34	− 2.7 [− 4.3 to − 1.1]	0.010	− 1.18
EX	2.8 (2.6)	2.4 (2.7)	1.3 (1.2)				TC vs. CON:	− 2.2 [− 3.9 to − 0.6]	0.052	− 0.91	− 3.3 [− 5.0 to − 1.6]	0.004	− 1.43
TC	3.2 (2.8)	1.3 (2.5)	0.9 (1.7)				TC vs. EX:	− 1.4 [− 3.0 to 0.3]	0.214	− 0.56	− 0.6 [− 2.3 to 1.1]	0.663	− 0.25
*Depression score*													
CON	3.0 (3.6)	3.2 (3.7)	3.4 (3.6)	0.005	0.137	0.119	EX vs. CON:	− 1.9 [− 3.4 to − 0.4]	0.030	− 0.75	− 2.8 [− 4.4 to − 1.3]	0.006	− 1.10
EX	4.0 (3.5)	1.9 (2.4)	1.3 (1.6)				TC vs. CON:	− 2.6 [− 4.2 to − 0.9]	0.007	− 0.95	− 2.7 [− 4.3 to − 1.1]	0.006	− 1.05
TC	3.9 (3.7)	1.2 (2.1)	1.3 (1.3)				TC vs. EX:	− 0.7 [− 2.3 to 1.0]	0.703	− 0.20	0.1 [− 1.5 to 1.7]	0.986	0.05
**Health-related quality of life**													
**12-item Short Form Survey**													
*Physical component summary*													
CON	48.2 (6.7)	45.2 (9.7)	42.2 (12.6)	0.002	0.012	0.010	EX vs. CON:	4.4 [− 0.8 to 9.6]	0.299	0.65	7.4 [2.2 to 12.6]	0.049	0.90
EX	45.4 (9.3)	47.8 (7.4)	47.8 (5.8)				TC vs. CON:	5.1 [− 0.4 to 10.6]	0.250	0.80	10.3 [4.9 to 15.8]	0.008	1.27
TC	43.6 (10.3)	47.3 (6.0)	49.5 (6.9)				TC vs. EX:	0.7 [− 4.7 to 6.2]	0.842	0.15	2.9 [− 2.5 to 8.4]	0.479	0.36
*Mental component summary*													
CON	57.4 (5.3)	55.4 (8.8)	55.1 (7.6)	0.255	0.186	0.252	EX vs. CON:	− 1.0 [− 6.4 to 4.3]	0.958	0.14	− 0.1 [− 5.4 to 5.3]	0.992	0.31
EX	53.7 (8.3)	52.7 (6.6)	53.4 (6.7)				TC vs. CON:	2.5 [− 3.2 to 8.2]	0.958	0.79	1.8 [− 3.9 to 7.5]	0.958	0.67
TC	51.2 (9.3)	55.1 (5.5)	54.0 (8.4)				TC vs. EX:	3.5 [− 2.1 to 9.1]	0.958	0.64	1.8 [− 3.8 to 7.4]	0.958	0.36
**Sleep**													
**Pittsburgh Sleep Quality Index**													
CON	5.3 (3.6)	6.3 (3.4)	5.7 (2.8)	0.118	0.453	0.230	EX vs. CON:	− 0.4 [− 2.2 to 1.5]	0.842	− 0.17	− 1.0 [− 2.8 to 0.8]	0.587	− 0.25
EX	5.2 (5.1)	5.8 (4.9)	4.6 (4.4)				TC vs. CON:	− 2.6 [− 4.5 to − 0.7]	0.137	− 0.70	− 2.0 [− 3.9 to − 0.1]	0.232	− 0.53
TC	5.7 (4.7)	4.0 (3.4)	4.0 (3.5)				TC vs. EX:	− 2.2 [− 4.1 to − 0.3]	0.191	− 0.53	− 1.0 [− 2.9 to 0.9]	0.587	− 0.28

All values are expressed as mean (SD). Generalized estimating equations with baseline measurement as a covariate was used to analyze the data. Pairwise comparisons were performed using closed test procedure with Holm-Bonferroni correction. *CON* Control group, *EX* Conventional exercise group, *TC* Tai Chi group.

## Discussion

Given the current available evidence that shows Tai Chi and conventional exercise induce changes in the brain through different mechanisms^16^ that lead to different structural and functional brain adaptions^19,20^, it is conceivable that these two exercise modalities confer different effects on global cognitive function and on specific cognitive domains. The present study directly compared the effects of the two exercise modalities on improving global cognitive function and on particular cognitive domains. After 12 weeks of intervention, Tai Chi, but not conventional exercise, provoked a clinically relevant improvement in global cognitive function (i.e., a minimum increase in the score of MoCA-HK by 4 points compared to baseline^33–35^). Both exercise groups exhibited significantly larger improvements in global cognitive function compared with the control but it is worth-noting that the improvement was more profound in the Tai Chi group. Upon completion of the 24-week intervention, both intervention groups manifested a clinically relevant improvement in global cognitive function and the improvements were significantly larger than that of the control group. Tai Chi led to a more evident improvement in global cognitive function than conventional exercise but such difference did not reach statistical significance. Furthermore, both interventions improved executive function, cognitive flexibility, long-term memory, short term memory, and attention after 24 weeks. Tai Chi improved cognitive flexibility more evidently and rapidly than conventional exercise, as indicated by a reduction in the TMT Part B/A ratio score. On the other hand, conventional exercise showed earlier improvements on attention than Tai Chi. Our data in line with existing reports that both interventions can improve cognitive function. The present findings have demonstrated the differential effects of different exercise modalities on global cognitive function and cognitive flexibility, but not memory.

The practical guidelines of the American Association of Neurology suggest a 6-month exercise program with a frequency of two sessions weekly can improve cognitive function in patients with MCI, but there are no specific recommendations on the exercise modality^5^. Here, we showed that conventional exercise and Tai Chi significantly improved global cognitive function after only 12 weeks of the intervention, which could be ascribed to the more frequent training sessions in our protocol. At the mid-assessment, Tai Chi resulted in more profound improvements on global cognitive function compared with conventional exercise. Moreover, Tai Chi, but not conventional exercise, showed an increase in MoCA-HK score that exceeded the defined score (i.e., 4 points) for a minimal clinically important difference^33–35^. Tai Chi elicited more rapid clinically relevant improvements on global cognitive function than conventional exercise, suggesting Tai Chi might benefit older adults with MCI with an earlier amelioration of the cognitive decline and would prevent related daily function impairments. After 24 weeks of intervention, both exercise modalities induced clinically relevant improvements in global cognitive function, which supports the recommendation of a 6-month exercise intervention period by the American Association of Neurology. Risk factors including poor sleep, depression, anxiety, and metabolic abnormalities are known to increase the risk of progression from MCI to dementia^24^. In line with the existing data, we demonstrated that the two exercise modalities could attenuate depression and anxiety scores in HADS. The beneficial effects of conventional exercise and Tai Chi on sleep is also well documented^55^. Our observation that neither intervention led to improved sleep quality may be due to the low baseline PSQI scores. Taken together, both exercise interventions were able to enhance cognitive function, making them promising interventions for preventing cognitive decline and possibly reducing the risk of progression from MCI to dementia. Although the differences in the improvements on global cognitive function between the two intervention groups became less evident after 24 weeks of intervention, Tai Chi tended to show more profound improvements. Further studies with larger sample sizes will be needed to substantiate the superior effectiveness and long-term benefits of Tai Chi compared with conventional exercise.

Notably, in the mid-assessment, TC showed a greater improvement in global cognitive function and an early improvement in Trail Making Test Part B/A ratio score, whereas EX showed an earlier improvement in the completion time of the Victoria Stroop Test under the interference condition. This data supports the notion that conventional exercise and Tai Chi manifest different effects on global cognitive function and cognitive domains of interest. Indeed, the two types of exercise differ in the mode of training. Conventional exercise emphasizes fitness training with simple repetitive movements, whereas Tai Chi emphasizes motor training through the practice of specific body movements with the incorporation of meditation^16^. Unlike fitness training that was found to improve global cognition by enhancing cardiorespiratory fitness, motor training has been shown to induce task-specific changes in the brain^56^. Therefore, the two exercise modalities induce specific brain adaptations by distinct mechanisms, which might account for our observations on the affected cognitive domains and different improvement rates. It should be noted that TMT requires cooperation of several cognitive domains^57^. The Part B/A ratio score provides a relatively purer measure of cognitive flexibility, a sub-domain of executive function, as it has reduced possible interference from other cognitive domains^46,47^. In line with a recent study, TC provoked a more profound improvement in cognitive flexibility than EX at both assessments, indicated by a larger decrease in the Part B/A ratio score^20^. Given that the physical activity intensity was comparable between the two exercise modalities in the present study, the greater improvements on cognitive flexibility induced by Tai Chi could be ascribed to its specific motor training and added meditation element. Cognitive flexibility refers to the ability to switch between different mental sets, thoughts, tasks, or strategies^58^, and is required for task-switching and multi-tasking^59^. Conventional exercises largely consist of simple, repetitive movements, whereas specific motor planning is necessary to ensure the smooth execution of successive complex movements in Tai Chi. Besides, previous studies showed that meditation alone is sufficient to enhance task-switching performance and cognitive flexibility^60,61^. This supports the incorporation of meditation and the cognitive demands of motor planning during Tai Chi practice to facilitate the improvements on cognitive flexibility. Taken together, intricate motor training in conjunction with meditation in Tai Chi might lead to earlier and more significant improvements on cognitive flexibility. Considering that each executive function subdomain is associated with a specific computation among the execution function-related brain regions^45^, we speculate that Tai Chi may enhance the specific computation of the brain regions related to cognitive flexibility in a more profound manner than conventional exercise, whereas the two exercise modalities induced similar enhancements of the specific computations of the brain regions related to other executive function subdomains. This may account for the more evident improvement in cognitive flexibility in the Tai Chi group, but similar improvements in other executive function subdomains induced by Tai Chi and conventional exercise observed in this study. However, further studies are needed to confirm this notion. At post-assessment, both exercise modalities improved all the assessed cognitive domains, except the language domain assessed by verbal fluency. Although we found that Tai Chi improved the TMT Part B/A ratio score and conventional exercise reduced the completion time of the Victoria Stroop Test at mid-assessment, it should be noted that the improvements in the other neurocognitive tests (including length of Digit Span Forward, TMT Part B and Part B-A difference scores) across both intervention groups were observed only after the completion of the 24-week interventions. Collectively, our data are congruent with the documented guidelines and suggests that 24 weeks of exercise intervention is required to confer significant improvements in any given cognitive domain regardless of the exercise modality.

Although Tai Chi provoked more prominent improvement on global cognitive function than conventional exercise, there were no significant differences in the assessed cognitive domains between the two intervention groups except for cognitive flexibility. However, this single significant change might not be sufficient to explain the superiority of Tai Chi over conventional exercise on improving global cognitive function. Contrary to previous observations in healthy adults, the improvements in memory and the executive function subdomains besides cognitive flexibility induced by Tai Chi and conventional exercise were not significantly different in older adults with MCI. Nevertheless, it is noteworthy that the effect sizes of Tai Chi on task-switching, executive control, and long term memory, measured by TMT Part B, TMT Part B-A minus score, and 30-min Delay recall Test, respectively, were larger than the conventional exercise counterparts, with small to moderate differences in magnitude (i.e., 0.30–0.49). Although Tai Chi appear to modulate these domains in a more pronounced manner compared with conventional exercise, the small sample sizes possibly prevented these improvements from reaching statistical significance. Both memory and executive function consist of an array of subdomains, but how these subdomains are modulated by the two exercise modalities remains to be deciphered. Moreover, as only executive function, memory, attention and language were assessed and reported as secondary outcomes, we cannot exclude the possibility that the other cognitive domains not assessed in this pilot study might contribute to more profound improvements in global cognitive function induced by the Tai Chi intervention. Notably, executive function, memory, and orientation have a larger weight relative to other assessed domains in MoCA-HK. Therefore, alterations in these cognitive domains likely lead to a considerable change in the MoCA-HK score due to their inherent and significant impact on the test^62,63^, which should be considered when interpreting the results. Our current findings will need to be validated in future studies with an adequate sample size, and the subdomains of executive function and memory should be comprehensively assessed along with the other cognitive domains not studied in the present work.

There are some limitations in the present study that need to be considered when interpreting the results. First, participants were advised to avoid gathering in groups because of the COVID-19 pandemic. Six off-site sessions were organized for the conventional exercise training, which required self-practicing while following the instructions of the respective teaching videos. Notably, we observed no significant differences in both the effectiveness of the instruction delivery and perceived exertion between video and face-to-face sessions (Supplementary Table 3). Therefore, we believe that the face-to-face and video sessions are equally effective. Second, this study excluded individuals with major diseases to avoid confounding factors on cognitive impairment that may be due to an underlying illness. However, we cannot rule out the possibility that there might be conditions that remained undiagnosed at the time of recruitment. Third, social activities are beneficial to cognitive function^64–66^. Participants in the intervention groups are exposed to interactions with the instructors and their fellow participants, thereby contributing to the observed improvement in cognitive function. However, the social interaction level of participants in the control group was not examined. Nonetheless, this did not affect the interpretation of the differences between the two exercise interventions. Fourth, it has been demonstrated in animal models that environmental enrichment is beneficial to cognitive function. A trip to the intervention venue may possibly increase the exposure of the environmental stimuli, both visual and auditory, and contributed to the observed larger improvement in cognitive function in the intervention groups compared with the control group. However, this should not affect the interpretation of the difference in effectiveness between the two intervention groups. Fifth, this study assessed only four cognitive domains (memory, attention, executive function, and language) out of the six key cognitive domains (the above four plus perceptual-motor function and social cognition) defined by Diagnostic and Statistical Manual of Mental Disorders, Fifth Edition^67^. Although we did not directly measure perceptual-motor function, static and dynamic balance were assessed by Single Leg Stand Test and Five Times Chair Stand Test, respectively^68^. Considering that static and dynamic balance is associated with perceptual-motor function^69^, a corresponding improvement in perceptual function will likely accompany improvements in static and dynamic balance in both intervention groups. However, improvements in balance ability may not fully address the effects of conventional exercise and Tai Chi on perceptual-motor function. Further in-depth studies and direct assessments are needed to evaluate the differences between these two exercise modalities on improving these domains. Sixth, our preliminary data obtained after 12 weeks of intervention support the notion that these two exercise modalities manifest different efficacies on global cognitive function and cognitive flexibility. However, although the 95% confident interval of the adjusted mean difference of the improvement in global cognitive function induced by Tai Chi and conventional exercise after the 24-week intervention did not contain zero, such difference did not reach statistical significance, which is likely ascribed to the small sample size. Further studies with an adequate sample size are needed to confirm the differences in long-term benefits between the two exercise modalities. Moreover, studies dissecting the respective underlying mechanisms of Tai Chi and conventional exercise on improving cognitive function and the respective changes induced in the brain are warranted to understand the beneficial effects of the two exercise modalities thoroughly.

In conclusion, both conventional exercise and Tai Chi improved global cognitive function and the performance of all the tested cognitive domains except for language after 24 weeks of intervention. Tai Chi conferred clinically relevant improvement on global cognitive function and improved cognitive flexibility more quickly and as early as after 12 weeks of intervention, whereas conventional exercise led to more rapid improvements in attention. These findings indicate the two exercise modalities have different effects on improving global cognitive function and cognitive domains. Compared with conventional exercise, the more immediate improvements in cognitive performance by Tai Chi training might benefit older adults with MCI, however, further investigations are needed to compare the effects of the two exercise modalities in a long-term intervention setting.

## Supplementary Information

Supplementary Information.
