# Supplementary material for: Tai Chi versus conventional exercise for improving cognitive function in older adults: a pilot randomized controlled trial
Source: Sci Rep. 2022 May 25;12:8868. doi: 10.1038/s41598-022-12526-5 (PMC9131984; doi:10.1038/s41598-022-12526-5)
Supplement: Supplementary file 1 — Supplementary Information. [file 41598_2022_12526_MOESM1_ESM.docx]

**Supplementary Table 1. Training Protocol of Convention Exercise Intervention**

| Session | Conventional exercise intervention |
| --- | --- |
| 1-36 | Static Stretching (approximately last for 10 minutes)   - Neck lateral flexion (20 seconds for each side, 2 sets) - Anterior cross-arm stretch (20 seconds for each side, 2 sets) - Behind the neck triceps stretch (20 seconds for each side, 2 sets) - Standing quadriceps stretch with chair assist (20 seconds for each side, 2 sets) - Seated toe touch stretch (20 seconds for each side, 2 sets) - Wall calf stretch (20 seconds for each side, 2 sets)   Muscle-strengthening exercise (approximately last for 20 minutes, 1 minute rest between set)   - Standing lateral raise with elastic band (15 repetitions per set, 2 sets) - Standing arm curl with elastic band (15 repetitions per set, 2 sets) - Squat with chair assist (15 repetitions per set, 2 sets) - Standing leg curl with chair assist (15 repetitions per set, 2 sets) - Calf raise with chair assist (15 repetitions per set, 2 sets)   Aerobic exercise (approximately last for 20 minutes, 30 seconds rest between set)   - Stepping with air shoulder press (30 seconds per set, 3 sets, moderate pace) - Stepping with arm swing (30 seconds per set, 3 sets, moderate pace) - Stepping with punching (30 seconds per set, 3 sets, moderate pace) - Stepping with arm abduction (30 seconds per set, 3 sets, moderate pace) - Stepping with arm curl (30 seconds per set, 3 sets, moderate pace) - Stepping with shoulder rotation (30 seconds per set, 3 sets, moderate pace)   Static Stretching (approximately last for 10 minutes)   - Neck lateral flexion (20 seconds for each side, 2 sets) - Anterior cross-arm stretch (20 seconds for each side, 2 sets) - Behind the neck triceps stretch (20 seconds for each side, 2 sets) - Standing quadriceps stretch with chair assist (20 seconds for each side, 2 sets) - Seated toe touch stretch (20 seconds for each side, 2 sets) - Wall calf stretch (20 seconds for each side, 2 sets) |
| 37-72 | Static Stretching (approximately last for 10 minutes)   - Neck lateral flexion (20 seconds for each side, 2 sets) - Anterior cross-arm stretch (20 seconds for each side, 2 sets) - Behind the neck triceps stretch (20 seconds for each side, 2 sets) - Standing quadriceps stretch with chair assist (20 seconds for each side, 2 sets) - Seated toe touch stretch (20 seconds for each side, 2 sets) - Wall calf stretch (20 seconds for each side, 2 sets)   Muscle-strengthening exercise (approximately last for 20 minutes, 1 minute rest between set)   - Standing lateral raise with elastic band (20 repetitions per set, 2 sets) - Standing arm curl with elastic band (20 repetitions per set, 2 sets) - Squat with chair assist (20 repetitions per set, 2 sets) - Standing leg curl with chair assist (20 repetitions per set, 2 sets) - Calf raise with chair assist (20 repetitions per set, 2 sets)   Aerobic exercise (approximately last for 20 minutes, 30 seconds rest between set)   - Stepping with air shoulder press (30 seconds per set, 3 sets, hands holding with 0.5 kg dumbbell ,moderate pace) - Stepping with arm swing (30 seconds per set, 3 sets, hands holding with 0.5 kg dumbbell ,moderate pace) - Stepping with punching (30 seconds per set, 3 sets, hands holding with 0.5 kg dumbbell ,moderate pace) - Stepping with arm abduction (30 seconds per set, 3 sets, hands holding with 0.5 kg dumbbell ,moderate pace) - Stepping with arm curl (30 seconds per set, 3 sets, hands holding with 0.5 kg dumbbell ,moderate pace) - Stepping with shoulder rotation (30 seconds per set, 3 sets, hands holding with 0.5 kg dumbbell ,moderate pace)   Static Stretching (approximately last for 10 minutes)   - Neck lateral flexion (20 seconds for each side, 2 sets) - Anterior cross-arm stretch (20 seconds for each side, 2 sets) - Behind the neck triceps stretch (20 seconds for each side, 2 sets) - Standing quadriceps stretch with chair assist (20 seconds for each side, 2 sets) - Seated toe touch stretch (20 seconds for each side, 2 sets) - Wall calf stretch (20 seconds for each side, 2 sets) |

**Supplementary Table 2. Training Protocol of Tai Chi Intervention**

| Session | Tai Chi intervention |
| --- | --- |
| 1 | - 10 min Introduction of Tai Chi, including the history, theory and basic working principal. - 15 min standing pose meditation and Tai Chi relaxation exercise - 25 min basic technique of weight shifting and walking - 10 min standing pose meditation and Tai Chi relaxation exercise |
| 2 | - 10 min standing pose meditation and Tai Chi relaxation exercise - 20 min basic technique of weight shifting and walking - 20 min learn and practice “starting pose of the 24 forms” - 10 min standing pose meditation and Tai Chi relaxation exercise |
| 3 | - 10 min standing pose meditation and Tai Chi relaxation exercise - 20 min learn “part the wild horse's mane“ - 20 min practice “starting pose of the 24 forms” and “part the wild horse's mane” - 10 min standing pose meditation and Tai Chi relaxation exercise |
| 4 | - 10 min standing pose meditation and Tai Chi relaxation exercise - 25 min practice “starting pose of the 24 forms” and “part the wild horse's mane” - 15 min learn” white crane spreads its wings - 10 min breathing and relaxation exercise |
| 5 | - 10 min standing pose meditation and Tai Chi relaxation exercise - 40 min practice “starting pose of the 24 forms”, ”part the wild horse's mane” and “white crane spreads its wings” - 10 min breathing and relaxation exercise |
| 6 | - 10 min standing pose meditation and Tai Chi relaxation exercise - 20 min practice “starting pose of the 24 forms”, ”part the wild horse's mane” and “white crane spreads its wings” - 20 min learn “brush knee and twist step” - 10 min breathing and relaxation exercise |
| 7 | - 10 min standing pose meditation and Tai Chi relaxation exercise - 40 min practice previously learnt forms - 10 min breathing and relaxation exercise |
| 8 | - 10 min standing pose meditation and Tai Chi relaxation exercise - 30 min practice previously learnt forms - 10 min learn “playing the lute” - 10 min breathing and relaxation exercise |
| 9 | - 10 min standing pose meditation and Tai Chi relaxation exercise - 40 min practice previously learnt forms - 10 min breathing and relaxation exercise |
| 10 | - 10 min standing pose meditation and Tai Chi relaxation exercise - 20 min practice previously learnt forms - 20 min learn “reverse reeling forearm” - 10 min breathing and relaxation exercise |
| 11 | - 10 min standing pose meditation and Tai Chi relaxation exercise - 40 min practice previously learnt forms - 10 min breathing and relaxation exercise |
| 12 | - 10 min standing pose meditation and Tai Chi relaxation exercise - 40 min practice previously learnt forms - 10 min breathing and relaxation exercise |
| 13 | - 10 min standing pose meditation and Tai Chi relaxation exercise - 40 min learn “grasp sparrow's tail” - 10 min breathing and relaxation exercise |
| 14 | - 10 min standing pose meditation and Tai Chi relaxation exercise - 40 min practice previously learnt forms - 10 min breathing and relaxation exercise |
| 15 | - 10 min standing pose meditation and Tai Chi relaxation exercise - 20 min learn “single whip” - 20 min practice previously learnt forms - 10 min breathing and relaxation exercise |
| 16 | - 10 min standing pose meditation and Tai Chi relaxation exercise - 40 min practice previously learnt forms - 10 min breathing and relaxation exercise |
| 17 | - 10 min standing pose meditation and Tai Chi relaxation exercise - 20 min learn “wave hands like clouds” - 20 min practice previously learnt forms - 10 min breathing and relaxation exercise |
| 18 | - 10 min standing pose meditation and Tai Chi relaxation exercise - 40 min practice previously learnt forms - 10 min breathing and relaxation exercise |
| 19 | - 10 min standing pose meditation and Tai Chi relaxation exercise - 20 min learn “high pat on horse” - 20 min practice previously learnt forms - 10 min breathing and relaxation exercise |
| 20 | - 10 min standing pose meditation and Tai Chi relaxation exercise - 40 min practice previously learnt forms - 10 min breathing and relaxation exercise |
| 21 | - 10 min standing pose meditation and Tai Chi relaxation exercise - 40 min learn “right heel kick” - 10 min breathing and relaxation exercise |
| 22 | - 10 min standing pose meditation and Tai Chi relaxation exercise - 40 min practice previously learnt forms - 10 min breathing and relaxation exercise |
| 23 | - 10 min standing pose meditation and Tai Chi relaxation exercise - 10 min learn “strike to ears with both fists” - 30 min practice previously learnt forms - 10 min breathing and relaxation exercise |
| 24 | - 10 min standing pose meditation and Tai Chi relaxation exercise - 40 min practice previously learnt forms - 10 min breathing and relaxation exercise |
| 25 | - 10 min standing pose meditation and Tai Chi relaxation exercise - 40 min learn “turn body and left heel kick” - 10 min breathing and relaxation exercise |
| 26 | - 10 min standing pose meditation and Tai Chi relaxation exercise - 40 min practice previously learnt forms - 10 min breathing and relaxation exercise |
| 27 | - 10 min standing pose meditation and Tai Chi relaxation exercise - 40 min learn “left lower body and stand on one leg” and “right lower body and stand on one leg” - 10 min breathing and relaxation exercise |
| 28 | - 10 min standing pose meditation and Tai Chi relaxation exercise - 40 min practice previously learnt forms - 10 min breathing and relaxation exercise |
| 29 | - 10 min standing pose meditation and Tai Chi relaxation exercise - 20 min practice previously learnt forms - 20 min learn “shuttle back and forth” - 10 min breathing and relaxation exercise |
| 30 | - 10 min standing pose meditation and Tai Chi relaxation exercise - 40 min practice previously learnt forms - 10 min breathing and relaxation exercise |
| 31 | - 10 min standing pose meditation and Tai Chi relaxation exercise - 20 min practice previously learnt forms - 20 min learn “needle at sea bottom” - 10 min breathing and relaxation exercise |
| 32 | - 10 min standing pose meditation and Tai Chi relaxation exercise - 40 practice previously learnt forms - 10 min breathing and relaxation exercise |
| 33 | - 10 min standing pose meditation and Tai Chi relaxation exercise - 20 min practice previously learnt forms - 20 learn “fan through back” - 10 min breathing and relaxation exercise |
| 34 | - 10 min standing pose meditation and Tai Chi relaxation exercise - 40 practice previously learnt forms - 10 min breathing and relaxation exercise |
| 35 | - 10 min standing pose meditation and Tai Chi relaxation exercise - 40 min learn “turn body, deflect, parry, and punch” - 10 min breathing and relaxation exercise |
| 36 | - 10 min standing pose meditation and Tai Chi relaxation exercise - 20 min practice previously learnt forms - 20 min learn “appears closed”, “cross hands” and “closing” - 10 min breathing and relaxation exercise |
| 37 – 72 | - 10 min standing pose meditation and Tai Chi relaxation exercise - 40 min practice previously learnt forms with refinement of posture, movement and meditation components - 10 min breathing and relaxation exercise |

The standing pose meditation was Zhan Zhuang, a common training method for traditional Chinese martial arts including Tai Chi, during which the participants were instructed to relax and stand with their leg slightly bent and hold their arms in a way to resemble hugging a big tree They were then asked to imagine hugging a tree while standing in a river with their body supported by the branches of tree and the water. The relaxation exercises involved stretching with a meditation element. There is a break of around 1 min for every 10 min of practice. The participants had learnt the completed 24-form form Yang style Tai Chi in first 36 sessions of the intervention. Given that a complete 24-form of Tai Chi last for approximately 5 minutes, the participants had performed 4 trials of the 24-form of Tai Chi in each training session of the later 36 training session.

**Supplementary Table 3. Comparison of the clarity of instructions and the perceived exertion between video and face-to-face sessions of the conventional exercise intervention**

|  | Face-to-face sessions | Video sessions | P-value |
| --- | --- | --- | --- |
| Clarity of instructions | 9.0 (1.0) | 8.5 (1.2) | 0.181 |
| Borg’s rate of perceived exertion | 13.0 (0.0) | 12.9 (0.5) | 0.773 |

All values are expressed as mean (standard deviation). Paired Wilcoxon signed-rank test was used to analyse the data.

Since participants were advised to avoid gathering in groups because of the COVID-19 pandemic, a total of 6 off-site sessions were organized for the conventional exercise training, which required self-practicing by following the instructions of the respective teaching videos. To verify whether the video sessions were equally effective as face-to-face sessions, participants were asked to rate the clarity of instructions in both face-to-face and video sessions on a 10-point scale (1 = most ambiguous and 10 = most concise). We did not observe any significant differences in the effectiveness of the instruction delivery (P=0.181). We also examined whether participants achieved the desired exercise intensity from self-practice training using Borg’s rating of perceived exertion on a 6-20 scale ^1^. Likewise, there were no significant differences in the perceived exertion between video and face-to-face sessions (P=0.773).

**Reference**

1 Chen, Y. L., Chen, C. C., Hsia, P. Y. & Lin, S. K. Relationships of Borg's RPE 6-20 scale and heart rate in dynamic and static exercises among a sample of young Taiwanese men. *Percept Mot Skills* **117**, 971-982, doi:10.2466/03.08.PMS.117x32z6 (2013).
